# Performance of multiplicom's BRCA MASTR Dx kit on the detection of *BRCA1* and *BRCA2* mutations in fresh frozen ovarian and breast tumor samples

**DOI:** 10.18632/oncotarget.12877

**Published:** 2016-10-25

**Authors:** Cindy Badoer, Céline Garrec, Dirk Goossens, Gillian Ellison, John Mills, Mélina Dzial, Hakim El Housni, Sarah Berwouts, Paola Concolino, Virginie Guibert-Le Guevellou, Capucine Delnatte, Jurgen Del Favero, Ettore Capoluongo, Stéphane Bézieau

**Affiliations:** ^1^ Laboratoire de Génétique Moléculaire, Clinique Universitaire de Bruxelles-Hôpital Erasme-Université Libre de Bruxelles (CUB-Erasme-ULB), Brussels, Belgium; ^2^ Institut de Biologie, Laboratoire de Génétique Moléculaire, Service de Génétique Médicale, CHU Nantes, Nantes, France; ^3^ Multiplicom N.V., Niel, Belgium; ^4^ AstraZeneca, Personalised Healthcare and Biomarkers, Alderley Park, Macclesfield, UK; ^5^ Laboratory of Clinical Molecular and Personalized Diagnostics, Foundation Policlinico Gemelli and Catholic University of Rome, Italy; ^6^ Molipharma and Giovanni Paolo II Foundation, Campobasso, Italy

**Keywords:** BRCA1-BRCA2, next generation sequencing, fresh frozen tumors, ovarian carcinoma, olaparib

## Abstract

Next-generation sequencing (NGS) has enabled new approaches for detection of mutations in the *BRCA1* and *BRCA2* genes responsible for hereditary breast and ovarian cancer (HBOC). The search for germline mutations in the *BRCA1* and *BRCA2* genes is of importance with respect to oncogenetic and surgical (bilateral mastectomy, ovariectomy) counselling. Testing tumor material for *BRCA* mutations is of increasing importance for therapeutic decision making as the poly ADP ribose polymerase (PARP) inhibitor, olaparib, is now available to treat patients with specific forms of ovarian cancer and *BRCA* mutations. Molecular genetics laboratories should develop reliable and sensitive techniques for the complete analysis of the *BRCA1* and *BRCA2* genes. This is a challenge due to the size of the coding sequence of the *BRCA1/2* genes, the absence of hot spot mutations, and particularly by the lower DNA quality obtained from Formalin-Fixed Paraffin-Embedded (FFPE) tissue. As a result, a number of analyses are uninterpretable and do not always provide a result to the clinician, limiting the optimal therapeutic management of patients. The availability of Fresh Frozen Tissue (FFT) for some laboratories and the excellent quality of the DNA extracted from it offers an alternative. For this reason, we evaluated Multiplicom's BRCA MASTR Dx assay on a set of 97 FFT derived DNA samples, in combination with the MID for Illumina MiSeq for *BRCA1* and *BRCA2* mutation detection. We obtained interpretable NGS results for all tested samples and showed > 99,7% sensitivity, specificity and accuracy.

## INTRODUCTION

Next Generation sequencing (NGS) is used in routine testing for germline mutations that cause rare diseases or hereditary cancers. Different laboratories have implemented NGS to analyze *BRCA1* and *BRCA2* [1, 2] or panels of candidate genes suspected as being involved in Hereditary Breast Ovarian Cancer (HBOC) [3, 4]. These panels include, in addition to the genes *BRCA1* and *BRCA2*, genes that were found to be associated with a breast cancer risk e.g. PALB2 carriers [5, 6]. NGS provides the advantage of being faster, cheaper, and more sensitive for detection of mosaicism [7] than approaches to screening such as High Resolution Melting (HRM) followed by Sanger sequencing [8]. Research into mutations in the *BRCA1* and *BRCA2* genes is undertaken to detect familial forms of predisposition to cancer of the breast and ovary, and also for personalized medicine approaches. It has been shown that patients with high grade serous ovarian cancer respond to treatment by poly (ADP-ribose) polymerase inhibitors. PARP is a protein involved in base excision repair (BER). The PARP inhibitors act by blocking the BER pathway and promote DNA double-strand breaks. In normal cells, these double-strand breaks are repaired by proteins involved in homologous recombination (HR) which includes the *BRCA1* and *BRCA2* proteins. The PARP inhibitors have shown their effectiveness in patients sensitive to cisplatin but in the stage of relapse and with a mutation in the *BRCA1* or *BRCA2* genes. The identification of mutations for personalized medicine indications requires a rapid testing for *BRCA1* and *BRCA2* mutations (4 to 8 weeks).

Hennessy et al [9] identified ovarian cancer patients with somatic BRCA pathogenic variants and proposed that such patients may derive therapeutic benefit from treatment with PARP inhibitors. The PARPi olaparib (Lynparza) [10] is currently approved for ovarian cancer patients with germline BRCA pathogenic variants in the US, but in the EU and most of the world, it is approved for patients with germline and somatic BRCA ones. It is therefore increasingly necessary for laboratories to search directly for mutations in the ovarian tumor, rather than only relying upon the testing of a blood sample. In the past, laboratories have already developed techniques to search for mutations in tumors but usually the mutations were concentrated in hot-spots within genes of interest (e.g. *KRAS*, *NRAS*, *CKIT*, *BRAF*, *PIK3CA,* etc.). There are NGS approaches to test the mutation hotspots in these genes individually or within gene panels. However, considering the NGS cost, these limited investigation in term of sequence target continue to be performed routinely with targeted techniques such as ARMS, Sanger sequencing or pyrosequencing [11, 12].

Since the *BRCA* genes are tumor suppressor genes and since the genes lack hot spot mutations, any mutation that results in a loss of function is potentially tumorigenic. Thus, to determine the mutation status of *BRCA1* or *BRCA2*, it is essential to sequence the complete genes (in practice coding sequence and intron/exon junctions).

This analysis is complicated by the large size of the coding sequences of the *BRCA1* and *BRCA2* genes ~20 000 bp). In addition, the tumor material available is most often in FFPE which presents several challenges. DNA extracted from FFPE is often degraded and limited, which may result in a number of cases that are unsuitable for a full analysis or can lead to uninterpretable result, even if some laboratories developed NGS with success on such DNA material [13, 14].

To ensure that patients eligible for treatment with the PARP inhibitors are not missed due to the testing methodology, some laboratories have chosen to test in parallel FFPE derived DNA extracted and peripheral blood derived DNA. This approach ensures a result for a putative germline *BRCA* mutation to enable a timely therapeutic decision to be made for PARP inhibitor treatment. DNA can be extracted from fresh frozen tumor tissue (FFT), which yields DNA of a quality and a quantity similar to that extracted from the blood. When FFT is available, it is therefore the material of choice for detection of both germline and somatic mutations by NGS based approaches.

Whether a *BRCA* mutation identified in FFT is of germline or somatic origin will subsequently need to be confirmed by analyzing a blood sample and this could be conducted using a simple hot spot test by Sanger sequencing, to confirm the presence of the specific mutation. This second analysis can be done later and is not necessary for the implementation of PARP inhibitor treatment.

In this multi-site study, we tested Multiplicom's BRCA MASTR Dx kit in 3 laboratories (Belgium, France, and Italy) followed by Illumina MiSeq sequencing. This report describes analytical performance characterization of the Multiplicom kit to detect *BRCA1* and *BRCA2* mutations in DNA isolated from fresh frozen ovarian and breast tumor tissues.

## RESULTS

**Figure 1:**
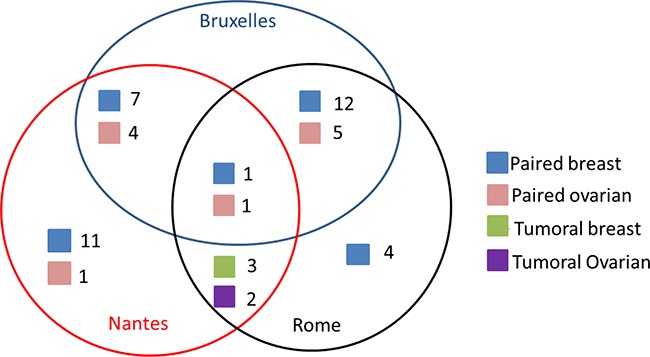
Scheme of the multicenter BRCA FFT study Each colored square represents a type of sample where blue= paired tumor and normal breast sample, red=paired tumor and normal ovarian sample, green= tumor breast sample and purple= tumor ovarian sample. The number next to each colored square represents the number of samples tested by each center (Brussels, Nantes and Rome).

The purpose of this multicenter study was to evaluate Multiplicom's BRCA MASTR Dx kit to detect clinically important variants in *BRCA1* and *BRCA2* in FFT derived ovarian and breast tumor samples. We tested the DNA extracted from 51 tumors of the breast or ovary in 3 laboratories (Figure 1). As expected and previously reported, the quality of the majority of the DNA samples extracted from FFT was excellent as compared to that of DNA extracted from FFPE [13].

### Coverage uniformity

The BRCA MASTR Dx kit covers all *BRCA1* and *BRCA2* coding regions, including 50 bp intron-exon junctions representing 20,635 bp of sequence.

It was important that sufficient depth of coverage was achieved to ensure the detection of potentially low frequency somatic variants with confidence. This needed to be balanced with maximizing the sequencing run capacity to analyze multiple samples per instrument run ensuring each sample was sufficiently represented in each run.

**Figure 2:**
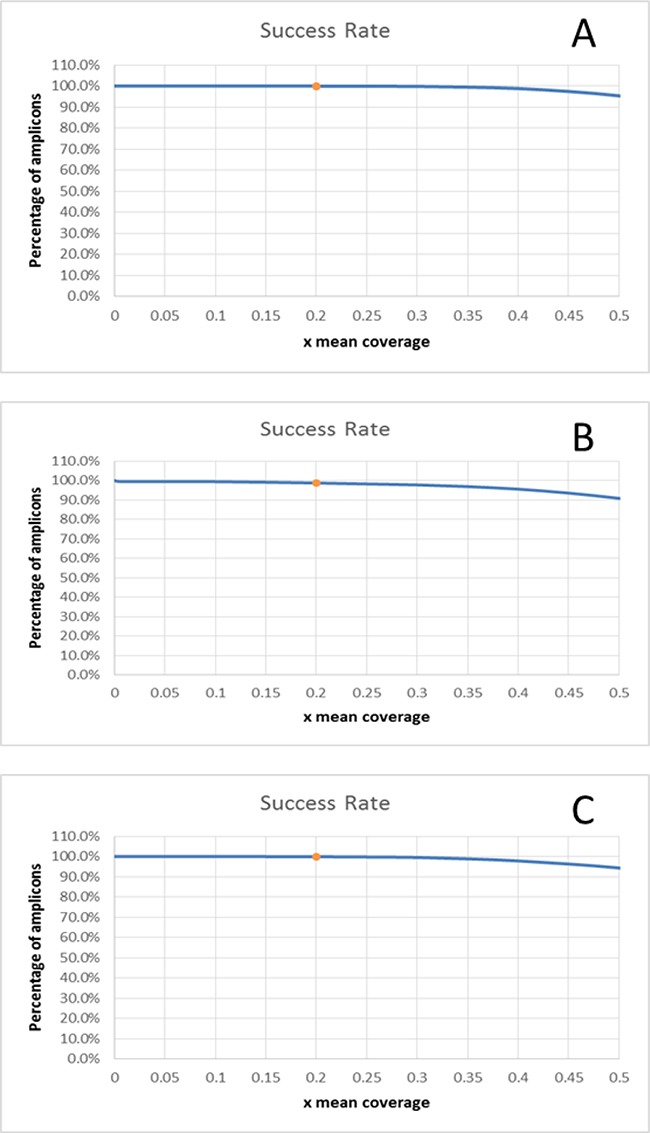
Coverage uniformity plots for the 3 centers A.100.0% > 0.2x mean (Nantes), B: 98.7% > 0.2x mean (Rome), C: 99.96% > 0.2x mean (Brussels).

We found that more than 99.9% of the targeted regions were covered at greater than 20% of the mean coverage in two laboratories and 99% was achieved by the third lab (Figure 2). This demonstrates that a comparable coverage uniformity has been reached for FFT samples compared/in regards to results previously obtained during the performance evaluation study on DNA extracted from blood (99.9% > 0.2x).

#### Variant detection

The SeqNext module of the Sequence Pilot software was used to identify all variants and alterations in *BRCA1* and *BRCA2*. The clinically significant variants (pathogenic- VUS) are listed in Table 1.

**Table 1:** The clinically significant variants (pathogenic - VUS) identified in *BRCA1* and *BRCA2* genes

Gene	Variant annotation (hg19)*	Average VAF	Clinical significance	Remark
BRCA2	chr13:g.[32932049A>GGGT]	0.44	Pathogenic Variants	
BRCA1	chr17:g.[41209079insG]	0.48	Pathogenic Variants	
BRCA1	chr17:g.[41228505C>A]	0.49	Pathogenic Variants	
BRCA1	chr17:g.[41244405delC]	0.49	Pathogenic Variants	
BRCA1	chr17:g.[41246443delC]	0.57	Pathogenic Variants	Tumor specific
BRCA1	chr17:g.[41258471A>G]	0.46	Pathogenic Variants	
BRCA2	chr13:g.[32890572G>A]	0.50	VUS	
BRCA2	chr13:g.[32900437T>C]	0.46	VUS	
BRCA2	chr13:g.[32936646T>C]	0.48	VUS	
BRCA2	chr13:g.[32968810T>C]	0.51	VUS	
BRCA2	chr13:g.[32972626A>T]	0.47	VUS	
BRCA2	chr13:g.[32972629A>C]	0.27	VUS	Tumor specific
BRCA1	chr17:g.[41222975C>T]	0.43	VUS	
BRCA1	chr17:g.[41223048A>G]	0.54	VUS	
BRCA1	chr17:g.[41243940C>T]	0.41	VUS	
BRCA1	chr17:g.[41244789A>G]	0.28	VUS	Tumor specific

* following the HGVS nomenclature

#### Analysis for analytical performance

As the entire coding regions of the BRCA genes (+50bp of the intronic junctions) were sequenced for 97 samples, 2001595 nucleotides were analyzed in total. Among those, 1001 variations have been detected (Table 2).

**Table 2:** Numbers of variants identified and classified regarding the clinical implication of the variant and the tissue they have been detected in^1^

Variant type	Breast tumor			Breast Total	Ovarian tumor			Ovarian Total	Total
	Tumor paired	Normal paired	Tumor sample		Tumor paired	Normal paired	Tumor sample		
Pathogenic mutation	3	3		6	3*	2	1	6	12
Variant of unknown significance	77*,†	76†	6	159	22*	21	3	46	205
Polymorphism	282	283†	14	579	97	97	11	205	784
Total	362	362	20	744	122	120	15	257	1001

1 * in this category a true somatic variant is found in the tumor paired sample in comparison to the normal sample

† presence of a variant classified as a false positive call.

**Figure 3:**
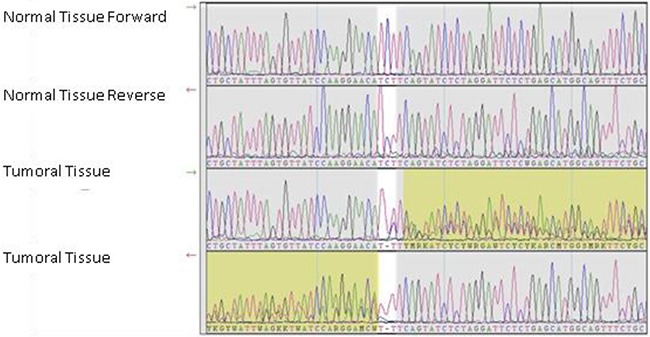
Sanger sequencing confirmatory run Electropherograms showing the hg19 chr17:g.41246443 position in the normal tissue and ovarian tumor tissue of a sample exhibiting a somatic frameshift mutation

In order to determine the analytical performance of the Multiplicom kit BRCA MASTR Dx on FFT samples (specificity, sensitivity and accuracy), all the sequenced nucleotides were classified as true positive, true negative, false positive or false negative variants (Table 3). Three somatic clinically relevant variants were discovered by virtue of being present in the tumor sample but not the matched normal samples. All of these were confirmed by Sanger DNA sequencing. These included a pathogenic variant (hg19 chr17:g.[41246443delC]) identified in an ovarian sample (Figure 3), and 2 VUS, one of which was found in a breast sample and the other in an ovarian sample.

**Table 3:** Classification of the variants detected as False Positive (FP), True Positive (TP), False Negative (FN), and True Negative (TN)

All target bases	2001595
FP	3
TP	998
FN	0
TN	2000594
Analytical Sensitivity	100% [99.6994% - 100%]
Analytical Specificity	99.9999% [99.9882% - 100%]
Analytical Accuracy	99.9999% [99.9882% - 100%]

Three of the detected variants were classified as false positive because they remained unconfirmed (allele frequency below 15%). Among them, two variants were detected in a paired breast normal sample by only one center with an unexpected low allelic frequency for germline variants (8% and 11%), while they were not identified by the two other labs. The third variant has been identified with an allelic frequency of 9% on a breast sample. This paired tumor sample has only been tested by one center. As the variant was not detected in the paired normal sample, this variant could be a tumor specific one; however we were not able to confirm it.

The specificity was calculated as TN/TN+FP, resulting in 99.9999% specificity (CI95 [99.988%-100%]), while the sensitivity, calculated as TP/TP+FN, was 100% (CI95 [99.699%-100%]).

Finally, the test accuracy was calculated as TP+TN/TP+FN+TN+FP, and was 99.9999% (CI95 [99.988%-100%]).

### Disproportionate allelic frequency

When comparing the variants identified in the tumor matched with the normal tissue, a difference of the allelic frequency of heterozygous variants in the tumor tissue was frequently observed. This phenomenon has been seen in 17 out of the 35 breast paired samples, and in 6 out of 11 ovarian paired samples. The allele frequency for these variants changed from ~50%, in the normal tissue, to disproportionate allele frequencies (ranging from 20% to 40% and 60% to 80%) in the tumor tissue. Interestingly, when this phenomenon was observed, all heterozygous variants of a gene behaved the same way. Conversely, the behavior of variants in both genes was not correlated. This may be accounted for by the observation that both BRCA genes must be inactivated to initiate tumorigenesis, and the second event (somatic) is typically through deletion of the other gene copy [15] (Welcsh, King 2001). This hypothesis was investigated further using Multiplicom's BRCA MAQ kit for copy number analysis.

### Copy number variation evaluation

A successful MAQ result was generated for 39 out 53 samples as these passed the quality score criteria for the method. The remaining samples did not pass QC and were not used for further analysis.

Twenty-seven samples did not show any gain or loss of copy number.

Nine samples showed CNVs of which three showed loss of *BRCA1*, three samples had complete loss of *BRCA2*, two cases presented a *BRCA1* duplication and one case a *BRCA2* duplication. Furthermore, three cases were found with partial loss or gain of the genes (Table 4).

**Table 4:** Allele copy number evaluated by MAQ kit

Allele status	*BRCA1*	*BRCA2*	*Total events (%)*
CNV within normal range	27	27	27 (69.2)
Complete Deleted (LOH)	3	3	6 (15.4)
Complete duplication (Gain)	2	1	3 (7.7)
Duplication exons 6-9	0	1	1 (2.6)
Deletion of exon20	0	2	2 (5.1)
**Total**	**5**	**7**	**12 (30.8)**

## DISCUSSION

In this study, we evaluated Multiplicom's BRCA MASTR Dx assay for the detection of *BRCA* mutations using DNA samples isolated from fresh frozen breast or ovarian cancer tissues. Multiplex PCR with the BRCA MASTR Dx assay, provided uniform amplification of the *BRCA1* and *BRCA2* genes on Illumina MiSeq sequencing and the assay was demonstrated to be sensitive and robust showing that all variants and mutations were detected down to a variant allele frequency of 15%.

This represents an alternative approach to the use of FFPE materials for the identification of *BRCA1* and *BRCA2* tumor mutations which may be of germline or somatic origin. FFPE is not the ideal starting material for molecular genetic testing as low yield of DNA and often degraded DNA is obtained from these samples. With poor quality FFPE-extracted DNA, efficient PCR amplification of DNA fragments is usually only achieved if they are shorter than 200bp. In theory, the quality of DNA extracted from FFT is as good as that of DNA extracted from blood: therefore, there is no need for specific technical adjustments of the assays used for this tumor derived material. The BRCA MASTR Dx kit from Multiplicom, which generates PCR fragments of ~480bp, can be used as such on FFT extracted DNA.

Bioinformatic analysis is also more straightforward from FFT derived DNA than of FFPE derived DNA mainly due to the reduction of sequencing artefacts. Moreover, it has been often reported that the number of variants identified in FFPE derived DNA is higher than those on FFT tissue as a result of the fixation of the tissue. Indeed, artefacts can lead to false-positive results due to the fact that the fixation of DNA with formalin solution can result in deamination of the cytosine residue leading to an uracil: deaminated cytosines are not correctly recognized by the Taq polymerase and an adenosine can be incorporated instead of a guanine when a deaminated cytosine is present in the DNA template. These artefacts often result as transitions from guanine to adenine (G>A) or cytosine to thymine (C>T) [16, 17, 18] (Bass et al, 2014; Srinivasan et al, 2002 and Williams et al, 1999). To eliminate false positive mutation interpretation, some laboratories add a uracil DNA glycosylase pretreatment to the protocol [19] (Serizawa et al).

In the present study our aim was to confirm the usefulness and applicability of molecular testing on fresh frozen tumor tissues. The analytical performance obtained using the BRCA MASTR Dx assay was excellent. The results obtained with respect to the specificity, sensitivity and accuracy corresponded to those observed when DNA had been extracted from blood, with each parameter being close to 100%. Furthermore, by comparing matched normal and tumor tissues, three variants were ascertained to be of somatic origin. Two were VUSs, and one was a pathogenic mutation. The identification of this pathogenic mutation, found only in the tumor sample, confirms the value of testing for tumor *BRCA1/2* mutations, and identifies patients with germline and somatic BRCA mutations who may be eligible for treatment with PARP inhibitor drugs such as olaparib.

Loss of heterozygosity (LOH) is hypothesized as an important mechanism by which the complete inactivation of BRCA1 and BRCA2 protein arises [20] (O Driscoll et al). In order to better decipher the role of some somatic VUS, quantitative allele evaluation should be helpful, given that these allele variants are not present in the normal tissue. In this regard, about 15% of our samples showed a complete deletion of the *BRCA1* or *BRCA2* genes, suggesting that LOH represents the most common event in tumor cells. As for germline assays, analysis of large rearrangements may represent a useful tool in particular for BRCA testing [21, 22] (Concolino P, CCLM and CCA 2014, see references listed).

In order to better discriminate between somatic and germline mutations, some laboratories test in parallel *BRCA* genes from tumor material and from normal tissue with the same BRCA MASTR Dx: this kit represents therefore an advantage, since different types of sample may be analyzed with the same complete pipeline, giving reliable results in terms of quality and performance of analysis.

## CONCLUSIONS

We showed that Multiplicom's BRCA MASTR Dx assay provides complete exon coverage of all coding sequences of *BRCA*1 and *BRCA*2 genes and combined with FFT derived DNA resulted in high sensitivity (≥99.7%), specificity (≥99.99%) and accuracy (≥99.99%).

Furthermore, we showed that FFT derived DNA is superior for diagnostic testing over FFPE derived DNA opening the way for multiple gene testing, above all for those involved in homologous recombination (HR) such as *RAD51* [23] (Pothuri B et al). Mutations in these genes are responsible for a ‘*BRCAness*’ or ‘*HRness*’, terms used to describe a phenotype of BRCA-linked ovarian cancer [24] (De Summa et al). Furthermore, formalin fixation used for FFPE material is known to alter the DNA and be responsible for subsequent false-positive results. In addition, the FFT also allows for searching epimutations more easily than the FFPE, as possible hypermethylation of the *BRCA1* gene, which also inactivates one allele of *BRCA*1 [25, 26] (Ruscito et al)(Jacot et al).

In conclusion, FFT can be used for routine screening for tumor *BRCA* mutations using the BRCA MASTR™ Dx assay enabling efficient and sensitive identification of germline and somatic BRCA mutations and therefore allow reliable identification of patients eligible for PARP inhibitor (olaparib) treatment.

## MATERIALS AND METHODS

### Samples

In total, 97 samples were used in this study: 13 fresh frozen ovarian tumor samples and 11 paired adjacent ovarian normal samples; 38 fresh frozen breast cancer samples and 35 paired adjacent breast normal samples. All samples were obtained from Asterand (Detroit, MI, USA) and collected with specific consents reviewed and approved by appropriate regulatory and ethical Authorities (For details: www.Asterand.com). For each sample, a hematoxylin and eosin stained thin section was histopathologically reviewed by the supplier to confirm the tumor type and sample adequacy from the supplier.

### DNA extraction from frozen tissue

A portion (approximately 100-200mg) of the tissue was cut from the frozen sample block and grounded under liquid nitrogen. DNA was extracted with the PureGene DNA purification kit (GentraSystems) according to the manufacturer's standard procedure.

DNA yields were measured spectrophotometrically and normalized to 100 ng/μl prior to use. The integrity of the extracted DNA (form normal and tumor tissues) was assessed by fragment analysis on a Labchip GX (Perkin Elmer). DNA integrity was considered “good” if the majority of DNA fragments were above 2000 bp and containing high molecular weight DNA, while “acceptable” if DNA fragments were above 2000 bp but with no or little high molecular weight DNA. Finally, DNA integrity was considered “poor” if all fragments were below 2000 bp.

### MAQ analysis

MAQ is a multiplex PCR based method for the detection and analysis of CNVs in a genomic region or gene of interest. This method consists of the simultaneous PCR amplification of specific fluorescently labelled target and reference sequences. The *BRCA1/2* MAQ kit includes two Master reaction mix (Plex A and Plex B) containing primers for 55 *BRCA1/2* amplicons target (TA) and 17 control amplicons (CA). The comparison of normalized intensities between the proband and reference individual results in a dosage quotient indicating the copy number of the target amplicon.

The BRCA MAQ v1.0 kit (Multiplicom) was used according to manufacturer's instructions. Briefly, after DNA quantification PCR reaction and fragment analysis steps were performed. Two multiplex PCR reactions (Plex A and Plex B) were setup for each patient: 20-50 ng of DNA was used in a final reaction volume of 15 μl, including 5 μl of Master reaction mix (Plex A or Plex B) and sterile water. The reaction mixes were cycled as follows: 10 min at 98°C, 23 cycles of 95°C 45 sec, 60°C 45 sec and 68°C 2 min, and a final step to 72°C for 10 minutes. Fragment analysis, was performed by adding 2 μl of the MAQ PCR product mixed with 0.3 μl of the size standard GS600 (Applied Biosystems Warrington, UK) and 10 μl of HiDi-Formamide (Applied Biosystems Warrington, UK). Ten DNAs were included in the analysis as reference controls. The products were size separated on 3500-Genetic Analyser (Applied Biosystems Warrington, UK) and the resulting data was analyzed using MAQ-S v2.0 analysis software (Multiplicom) to calculate the Dosage Quotient (DQ) for all test and control amplicons, quantifying the copy number.

The cut-offs for discriminating a possible heterozygous deletion, leading to LOH, and a heterozygous gain were respectively: 0.25<DQ<0.75 and 1.3<DQ<1.75.

### Multiplex PCR-based target amplification and MiSeq sequencing

Targeted amplification of all coding exons of *BRCA1* and *BRCA2* was performed using the BRCA MASTR Dx kit from Multiplicom as described by the manufacturer (http://www.multiplicom.com/products/brca-mastr-dx). Briefly, five multiplex PCR reactions were set up using 50 ng of DNA per reaction. The resulting amplicons of each multiplex PCR were diluted 1000 fold followed by a second round of universal PCR enabling tagging of the amplicons with sample specific MIDs and MiSeq sequencing adaptors. The resulting tagged amplicons were mixed per individual applying a predefined assay specific mixing scheme. Each amplicon library was subsequently purified from small residual DNA fragments (Agencourt AMPure beads, Beckman Coulter) and DNA concentration was determined using PicoGreen, Nanodrop (Thermo Scientific) or Qubit. These methods for DNA quantification can be alternatively used to measure the library concentration: nevertheless, since an equimolar pooling per sequencing run was needed, any Lab used the same DNA measure method for each run. These purified and individually tagged amplicon libraries were pooled in equimolar amounts, resulting in an amplicon pool or sequencing sample, which was then sequenced on the Illumina MiSeq sequencing platform using the v3 600 cycles chemistry according to the manufacturer's instructions.

### Sequencing data analysis and variant calling

Sequencing data were analyzed using the SeqNext module version 4.1.1 of the Sequence Pilot software (JSI Medical systems GmbH, Kippenheim, Germany) as described in Multiplicom's IFU021 (http://www.multiplicom.com/sites/default/files/ifu021_partiii_data_analysis_for_illumina_miseq_v131216.pdf). In short, FastQ files were uploaded and trimmed to remove primer sequences. Next, sequence reads were aligned to the targeted regions and variants called if the coverage was > 100x for variants in normal sample DNA and >500x for variants in tumor DNA. Variants were considered as true positive if they were: a) identified in both the normal and tumor tissue of a sample; or b) reproducibly detected in different laboratories; c) called in forward and reverse modality at established coverage (about 50% for any allele).

Due to the limited sensitivity of Sanger sequencing, only potential false positive calls with an allele frequency above 15% were confirmed by Sanger sequencing. Variants with low coverages, when not not confirmed by Sanger, were classified as false positive, due to the limitation of Sanger Sequencing.

Variants were classified according to UMD *BRCA* database [27] (Caputo et al.), Breast International Consortium (BIC) database, or LOVD database [28] (Vallee et al.).
